# Supplementary material for: Proteomic characterization of acute kidney injury in patients hospitalized with SARS-CoV2 infection
Source: Commun Med (Lond). 2023 Jun 12;3:81. doi: 10.1038/s43856-023-00307-8 (PMC10258469; doi:10.1038/s43856-023-00307-8)
Supplement: Supplementary file 1 — Description of Additional Supplementary Files [file 43856_2023_307_MOESM1_ESM.pdf]

Description of Additional Supplementary File

File Name: Supplementary Data 1

Description:

Table 1 Demographic and clinical comorbidities of patients hospitalized with COVID-19 in the discovery cohort separated by the development of AKI (stage 2 or 3) during their hospital course.

Table 2 Validation of AKI-associated proteins. Strength of association of proteins significantly associated with AKI (stage 2 or 3) in both the discovery (adjusted P <0.05) and validation cohort (P <0.05) are provided. Significance was determined by fitting a linear model adjusted for age, sex, history of CKD, and maximum oxygen requirement at the time of blood draw.

Table 3 Association of AKI-associated proteins with post-discharge eGFR. A mixed effects linear model adjusted for age, sex, history of CKD, baseline creatinine, AKI stage during the COVID admission and number repeated eGFR measurements was fit. Proteins significantly associated with eGFR (adjusted P <0.05) are provided.

File Name: Supplementary Data 2

Description: Proteins that were significantly associated with AKI stage 2 or 3 (adjusted P <0.05) in the discovery cohort are provided. Statistical significance was estimated by fitting a linear model adjusted for age, sex, history of CKD, and maximum oxygen requirement at the time of blood draw using the Limma R package53. Protein expression data was log2 transformed. P values were adjusted for multiple comparisons using the Bonferroni correction.

File Name: Supplementary Data 3

Description: Full data table detailing longitudinal eGFR values for discharged COVID patients.
Subject_ID is the individual patient ID.
Value is the raw value of serum creatinine collected at that instant.
eGFR is the estimated glomerular filtration rate (eGFR) calculated from the serum creatinine value for each timepoint listed in the resultinstant column.
