# Supplementary material for: Proteomic characterization of acute kidney injury in patients hospitalized with SARS-CoV2 infection
Source: Commun Med (Lond). 2023 Jun 12;3:81. doi: 10.1038/s43856-023-00307-8 (PMC10258469; doi:10.1038/s43856-023-00307-8)

Supplementary Material

Supplementary Table 1: Demographic and clinical comorbidities of patients in the validation cohort. Cases include patients who developed AKI stage 2 or 3 during hospitalization. Controls include patients who developed AKI stage 1 or did not develop AKI during hospitalization.

Characteristic	Developed AKI (stage 2 or 3) during hospitalization (N = 35)	AKI stage 1 or no AKI during hospitalization (N = 226)	P Value
**Age, mean (SD)**	68.4 (17.1)	70.2 (17.5)	0.534
**Male, n (%)**	14 (40%)	120 (53%)	0.203
**Ancestry, n (%)**			0.341
European	22 (63%)	151 (67%)	
East Asian	2 (6%)	22 (10%)	
African	5 (14%)	27 (12%)	
South Asian	1 (3%)	4(2%)	
Mixed American	1 (3%)	4(2%)	
Unknown	1 (3%)	0 (0%)	
Other	3 (9%)	18 (8%)	
**Comorbidities, n (%)**			
Atrial Fibrillation	5 (14%)	38 (17%)	0.811
Coronary Artery Disease	9 (26%)	32 (14%)	0.0859
Arterial Hypertension	23 (66%)	142 (63%)	0.851
Diabetes	15 (43%)	77 (34%)	0.344
Chronic Kidney Disease	10 (29%)	25 (11%)	0.0130
**Highest respiratory support, n (%)**			<0.001
Intubation	17 (49%)	30 (13%)	
Non-invasive ventilation (CPAP, BIPAP, high-flow cannula)	3 (9%)	23 (10%)	
Nasal cannula	5 (14%)	94 (42%)	
None of the above	10 (29%)	79 (35%)	

Supplementary Table 2: Distribution of the number of SomaScan timepoints per person in the discovery cohort.

Number of Somascan Time points	Number of patients
1	203
2	137
3	69
4	30
5	15
6	1
10	1

Supplementary Figure 1

Protein quantitative trait loci (pQTL) evidence for 62 AKI-associated proteins. Associations were taken from proteomic and genomic data measured using human plasma samples.

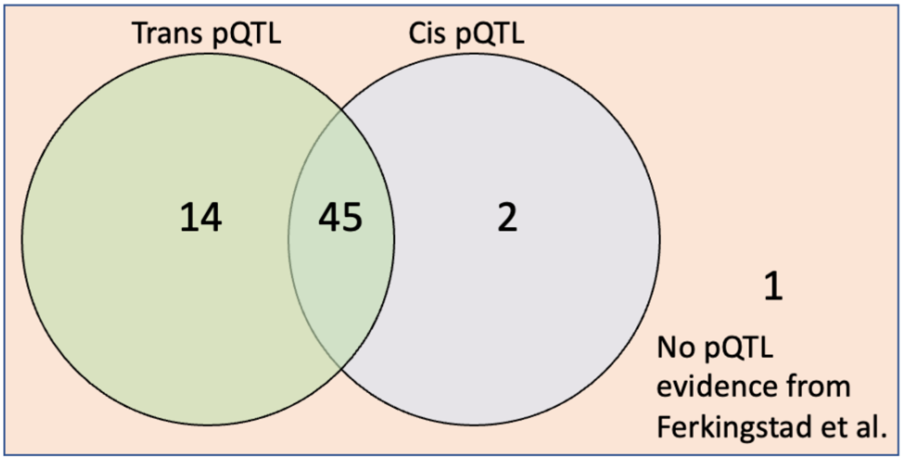

Supplementary Figure 2

Distribution of number of days after discharge for the first and last post-discharge eGFR measurements. All measurements were extracted from the EHR as part of routine clinical care.

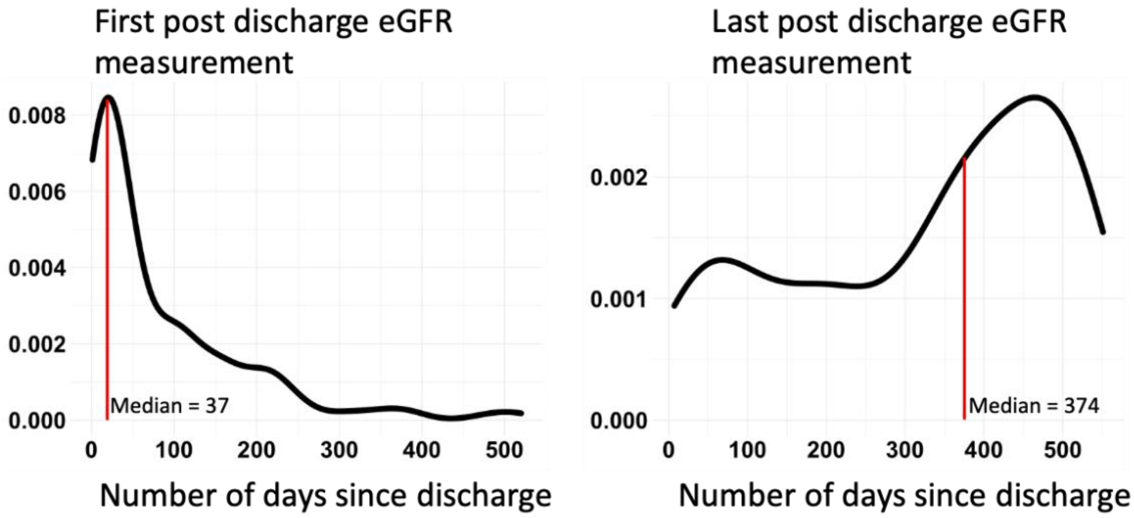

Supplement: Supplementary file 2 — Supplementary Information [file 43856_2023_307_MOESM2_ESM.pdf]
