# Supplementary material for: Proteomic characterization of acute kidney injury in patients hospitalized with SARS-CoV2 infection
Source: Commun Med (Lond). 2023 Jun 12;3:81. doi: 10.1038/s43856-023-00307-8 (PMC10258469; doi:10.1038/s43856-023-00307-8)
Supplement: Supplementary file 7 — Reporting Summary [file 43856_2023_307_MOESM7_ESM.pdf]

nature portfolio

Corresponding author(s): Girish Nadkarni, MD, MPH

Last updated by author(s): March 03 2023

Reporting Summary

Nature Portfolio wishes to improve the reproducibility of the work that we publish. This form provides structure for consistency and transparency in reporting. For further information on Nature Portfolio policies, see our Editorial Policies and the Editorial Policy Checklist.

Statistics

For all statistical analyses, confirm that the following items are present in the figure legend, table legend, main text, or Methods section.

n/a	Confirmed	
☐	☒	The exact sample size (n) for each experimental group/condition, given as a discrete number and unit of measurement
☐	☒	A statement on whether measurements were taken from distinct samples or whether the same sample was measured repeatedly
☐	☒	The statistical test(s) used AND whether they are one- or two-sided<br>*Only common tests should be described solely by name; describe more complex techniques in the Methods section.*
☐	☒	A description of all covariates tested
☐	☒	A description of any assumptions or corrections, such as tests of normality and adjustment for multiple comparisons
☐	☒	A full description of the statistical parameters including central tendency (e.g. means) or other basic estimates (e.g. regression coefficient) AND variation (e.g. standard deviation) or associated estimates of uncertainty (e.g. confidence intervals)
☐	☒	For null hypothesis testing, the test statistic (e.g. F, t, r) with confidence intervals, effect sizes, degrees of freedom and P value noted<br>*Give P values as exact values whenever suitable.*
☒	☐	For Bayesian analysis, information on the choice of priors and Markov chain Monte Carlo settings
☒	☐	For hierarchical and complex designs, identification of the appropriate level for tests and full reporting of outcomes
☐	☒	Estimates of effect sizes (e.g. Cohen's d, Pearson's r), indicating how they were calculated

Our web collection on statistics for biologists contains articles on many of the points above.

Software and code

Policy information about availability of computer code

Data collection	Our data was collected as part of a larger COVID study. We currently have a repository (linked to another paper https://www.nature.com/articles/s41467-020-17405-z#data-availability from our group) that includes data for this larger COVID-19 analysis cohort (https://adknowledgeportal.synapse.org/). Our patients were subsets of that COVID-19 cohort. We will include a link to the data in our final manuscript once it is uploaded prior to publication.
Data analysis	Code for analysis of data, models and code for post-processing of data is available at https://github.com/Nadkarni-Lab/aki_covid_proteomics

For manuscripts utilizing custom algorithms or software that are central to the research but not yet described in published literature, software must be made available to editors and reviewers. We strongly encourage code deposition in a community repository (e.g. GitHub). See the Nature Portfolio guidelines for submitting code & software for further information.

Data

Policy information about availability of data

All manuscripts must include a data availability statement. This statement should provide the following information, where applicable:

- Accession codes, unique identifiers, or web links for publicly available datasets
- A description of any restrictions on data availability
- For clinical datasets or third party data, please ensure that the statement adheres to our policy

Data Availability

The Data is available in Synapse syn35874390 here: https://www.synapse.org/#!Synapse:syn35874390. Access to the data and steps to process the clinical information to create the cohort is detailed on the site.

Validation cohort was The Biobanque Québécoise de la COVID-19 (BQC19) cohort. For individual-level data in BQC19, BQC19 received ethical approval from the Jewish General Hospital research ethics board (2020-2137) and the Centre hospitalier de l'Université de Montréal institutional ethics board (MP-02-2020-8929, 19.389). All participants gave informed consent.
More information on the plasma proteome from BQC19 can be viewed at https://www.mcgill.ca/genepi/mcg-covid-19-biobank. Access to the data of BQC19 can be obtained upon approval of requests via bqc19.ca

Human research participants

Policy information about studies involving human research participants and Sex and Gender in Research.

Reporting on sex and gender	Information on patient sex was collected as part of the data collection. The model was adjusted for Sex as one of the covariates.
Population characteristics	Table 1 of the Manuscript details the patient cohort characteristics for our study.
Recruitment	An overview of the discovery cohort selection process is provided in Fig 1. We prospectively enrolled patients hospitalized with COVID-19 between March 24, and August 26, 2020, at five hospitals of a large urban, academic hospital system in New York City, NY into a cohort as previously described34. The cohort enrolled (with informed consent) patients who were admitted to the health care system with a COVID-19 infection and had broad inclusion criteria without specific exclusion criteria. The Mount Sinai Institutional Review Board approved this study under a regulatory approval allowing for access to patient level data and biospecimen collection35. Peripheral blood specimens were collected at various points during the hospital admission for each patient.<br>The validation cohort included a prospective biobank from Quebec, Canada that enrolled patients hospitalized with COVID-19, as previously described29. Patients were recruited from the Jewish General Hospital and Centre Hospitalier de l'Université de Montréal. Peripheral blood specimens were collected at multiple time points after admission.
Ethics oversight	The IRB protocol and ethics oversight information is detailed in the cited manuscript. We have documented/cited this manuscript (for the same purpose/information) in our manuscript as well.: Del Valle, D.M., Kim-Schulze, S., Huang, HH. et al. An inflammatory cytokine signature predicts COVID-19 severity and survival. Nat Med 26, 1636–1643 (2020). https://doi.org/10.1038/s41591-020-1051-9

Note that full information on the approval of the study protocol must also be provided in the manuscript.

Field-specific reporting

Please select the one below that is the best fit for your research. If you are not sure, read the appropriate sections before making your selection.

☒ Life sciences ☐ Behavioural & social sciences ☐ Ecological, evolutionary & environmental sciences

For a reference copy of the document with all sections, see nature.com/documents/nr-reporting-summary-flat.pdf

Life sciences study design

All studies must disclose on these points even when the disclosure is negative.

Sample size	. Our total patient cohort for the study was 437 patients of which 71 were cases and the rest were controls. numbers allow for a power of >90% for effect sizes >1.9 and a power of ~70% at an effect size of 1.6. In addition, we have validated the top associations in an independent cohort..
Data exclusions	First, samples were collected during the hospital course of patients with confirmed COVID-19. However, the timepoints were not systematic due to logistical challenges during the peak of the COVID-19 pandemic and thus are not standardized between patients. Since a subset of patients had AKI at the time of admission, these patients were excluded from our analysis since specimens were collected after admission. Additionally, we did not include patients who developed AKI without COVID and were unable to determine whether COVID-AKI has unique proteomic markers compared to other forms of sepsis-AKI. Thus, our AKI cases may be biased towards less severe presentations.<br>We were not able to exclude individuals who were lost to follow-up or died because the data was extracted from an institutional EHR. Some patients accessed care at other hospitals after discharge. This remains a limitation as well. Finally, our cohort did not include autopsy or kidney biopsy specimens. Histopathological analysis of kidney specimens is necessary to determine the mechanism of AKI and whether viral particles are present in the kidney.
Replication	measured approximately 4496 plasma proteins by Somalogic proteomic assay in patients with COVID-19. The results were associated with the presence of AKI stage 2-3 and follow up eGFR as estimated by serum-creatinine. Findings were validated in an external, independent cohort
Randomization	Our total patient cohort for the study was 437 patients of which 71 were cases and the rest were controls.

Blinding | blinding was not relevant in this study because this was a case-control study.

Reporting for specific materials, systems and methods

We require information from authors about some types of materials, experimental systems and methods used in many studies. Here, indicate whether each material, system or method listed is relevant to your study. If you are not sure if a list item applies to your research, read the appropriate section before selecting a response.

Materials & experimental systems

n/a	Involved in the study
☒	☐ Antibodies
☒	☐ Eukaryotic cell lines
☒	☐ Palaeontology and archaeology
☒	☐ Animals and other organisms
☐	☒ Clinical data
☒	☐ Dual use research of concern

Methods

n/a	Involved in the study
☒	☐ ChIP-seq
☒	☐ Flow cytometry
☒	☐ MRI-based neuroimaging

Clinical data

Policy information about clinical studies

All manuscripts should comply with the ICMJE guidelines for publication of clinical research and a completed CONSORT checklist must be included with all submissions.

Clinical trial registration: *Provide the trial registration number from ClinicalTrials.gov or an equivalent agency.*

Study protocol: Del Valle, D.M., Kim-Schulze, S., Huang, HH. et al. An inflammatory cytokine signature predicts COVID-19 severity and survival. Nat Med 26, 1636–1643 (2020). https://doi.org/10.1038/s41591-020-1051-9

Data collection: Del Valle, D.M., Kim-Schulze, S., Huang, HH. et al. An inflammatory cytokine signature predicts COVID-19 severity and survival. Nat Med 26, 1636–1643 (2020). https://doi.org/10.1038/s41591-020-1051-9

Outcomes: We defined an AKI cohort using proteomic data acquired at the last available timepoint during the hospital course for all individuals. Patients who developed AKI after the last specimen collection timepoint were excluded. Controls were defined as individuals who developed AKI stage 1 or did not develop AKI during their hospital course.
